# Towards modifying the genetic predisposition for glaucoma: An overview of the contribution and interaction of genetic and environmental factors

**DOI:** 10.1016/j.mam.2023.101203

**Published:** 2023-07-08

**Authors:** Kelsey V. Stuart, Louis R. Pasquale, Jae H. Kang, Paul J. Foster, Anthony P. Khawaja

**Affiliations:** a NIHR Biomedical Research Centre, Moorfields Eye Hospital NHS Foundation Trust and UCL Institute of Ophthalmology, London, UK; b Department of Ophthalmology, Icahn School of Medicine at Mount Sinai, New York, NY, USA; c Department of Medicine, Brigham and Women’s Hospital, Harvard Medical School, Boston, MA, USA

**Keywords:** Glaucoma, Risk factors, Genetics, Gene-environment interactions, Review

## Abstract

Glaucoma, the leading cause of irreversible blindness worldwide, is a complex human disease, with both genetic and environmental determinants. The availability of large-scale, population-based cohorts and biobanks, combining genotyping and detailed phenotyping, has greatly accelerated research into the aetiology of glaucoma in recent years. Hypothesis-free genome-wide association studies have furthered our understanding of the complex genetic architecture underpinning the disease, while epidemiological studies have provided advances in the identification and characterisation of environmental risk factors. It is increasingly recognised that the combined effects of genetic and environmental factors may confer a disease risk that reflects a departure from the simple additive effect of the two. These gene-environment interactions have been implicated in a host of complex human diseases, including glaucoma, and have several important diagnostic and therapeutic implications for future clinical practice. Importantly, the ability to modify the risk associated with a particular genetic makeup promises to lead to personalised recommendations for glaucoma prevention, as well as novel treatment approaches in years to come. Here we provide an overview of genetic and environmental risk factors for glaucoma, as well as reviewing the evidence and discussing the implications of gene-environment interactions for the disease.

## 1. Introduction

Glaucoma comprises a heterogeneous group of disorders characterised by chronic progressive optic neuropathy and corresponding stereotypical visual field changes. The final common pathway for all forms of the disease is marked by retinal ganglion cell (RGC) degeneration and optic nerve fibre loss. Glaucoma is the leading cause of irreversible blindness worldwide, currently estimated to affect 76 million individuals aged 40–80 years, with projections rising to 112 million by 2040 (Tham et al., 2014). Globally, 2.1 million individuals are blind, and a further 4.2 million visually-impaired, as a result of the disease (Bourne et al., 2016).

Despite extensive research, the precise pathophysiological mechanisms underlying glaucomatous neurodegeneration remain unclear, although numerous hypotheses have been proposed (Stein et al., 2021). The biomechanical and vascular theories implicate intraocular pressure (IOP)-mediated mechanical stress and optic nerve head (ONH) vascular insufficiency, respectively, while a third theory posits a primary neurodegenerative component to the disease, especially when glaucomatous changes occur in the absence of raised IOP (normal tension glaucoma, NTG).

Glaucoma can be broadly categorised into two groups – open-angle glaucoma and angle-closure glaucoma – based on the configuration of the anterior chamber drainage angle. Both subtypes can occur as primary disease (>90% of cases) or secondary to an identifiable underlying mechanism (Quigley, 1996). In primary open-angle glaucoma (POAG) there is a normal anatomical drainage angle and no identifiable secondary cause for glaucoma (e.g., ocular pigment, exfoliation material, or inflammatory debris). POAG accounts for >80% of all glaucoma cases worldwide and is a highly complex disease, with both genetic and environmental determinants (Stein et al., 2021).

Well-established non-modifiable risk factors for POAG include older age, non-White ethnicity, and family history of glaucoma (Stein et al., 2021) – with the last two almost certainly reflecting some degree of genetic influence. Similarly, elevated IOP, the only known modifiable risk factor for glaucoma, is a heritable trait, with considerable overlap in the underlying genetic architecture of IOP and glaucoma (Khawaja et al., 2018). Numerous landmark interventional studies have proven the clinical benefit of lowering IOP on reducing the risk for the onset or progression of disease (Kass et al., 2002; Heijl et al., 2002), and this beneficial effect has also been demonstrated for NTG (Collaborative Normal-Tension Glaucoma Study Group, 1998), in which baseline IOP is within the normative population range, even before intervention. It is hypothesised that certain factors, including vascular dysregulation and abnormal pressure gradients across the lamina cribrosa, may render individuals with NTG more susceptible to IOP-mediated mechanical stress, resulting in glaucomatous damage at seemingly normal pressures (Killer and Pircher, 2018). Although all currently approved glaucoma interventions (including medication, laser, and surgery) work by lowering IOP, there is considerable interest in identifying other modifiable risk factors, which may complement existing treatment strategies or guide lifestyle recommendations.

## 2. Glaucoma genetics

Glaucoma is one of the most heritable of all complex human diseases (estimated *h*^2^, 0.70) (Wang et al., 2017), with first-degree relatives of individuals with glaucoma having an almost 10-fold greater lifetime risk of disease compared to the general population (Wolfs et al., 1998). In the paediatric setting, monogenetic mutations associated with primary congenital glaucoma are the most common cause of disease, accounting for a significant proportion of all childhood blindness (Lewis et al., 2017). Conversely, only a small proportion of POAG in adults (estimated to be <5%) is inherited in a Mendelian fashion (Stein et al., 2021). *MYOC* (myocilin) gene sequence variations give rise to the most common form, characterised by elevated IOP; while rare missense mutations in *OPTN* (optineurin) and copy number variations involving *TBK*-1 (TANK-binding protein 1) can cause familial NTG (Sears et al., 2019). These highly penetrant autosomal-dominant genetic mutations tend to have large biological effects, causing clinically severe early-onset disease (Wiggs and Pasquale, 2017). In this small subset of individuals, genetic testing of unaffected relatives may guide screening strategies, with a *MYOC* cascade genetic testing approach shown to identify at-risk individuals at a significantly younger age and with less severe disease than those presenting through traditional clinical referral pathways (Souzeau et al., 2017). Advances in genome-editing technology also holds the promise of curative precision medicine for these patients in the future. Disruption of mutant *MYOC* and its function using CRISPR-Cas9 resulted in lower IOP and prevented further glaucomatous damage in a mouse model of the disease (Jain et al., 2017a).

The genetics underpinning the vast majority of adult-onset POAG, however, is far more complex. In these cases, a multitude of genetic factors, each relatively common but of small individual effect, cumulatively contribute towards the risk of disease. In the last decade, hypothesis-free genome-wide association studies (GWAS) have driven the discovery of these common genetic determinants of glaucoma, with more than 100 POAG susceptibility loci reported to date (Choquet et al., 2020; Gharahkhani et al., 2021). This line of research has been greatly accelerated by the emergence of large-scale biobank-based cohorts and collaborative genetic consortia, the widespread availability of GWAS summary statistics to the scientific community, and advances in post-GWAS genetic analyses (Choquet et al., 2020). Despite this rapid progress, current knowledge of genome-wide significant (*P* < 5 × 10^−8^) single nucleotide polymorphisms (SNPs) explains less than 10% of the genetic contribution to POAG susceptibility, suggesting that additional variants are yet to be discovered (Craig et al., 2020).

To aid genetic discovery, the definitive case-control GWAS approach has been supplemented by the examination of heritable quantitative traits – termed endophenotypes – related to glaucoma. This approach, which includes analysis of IOP and optic disc parameters, is not reliant only on data from disease cases, but can instead leverage data from a healthy population by assessing the variation of an endophenotype across a spectrum of health and disease. In this way, population cohorts can contribute to analyses, greatly increasing sample size and power to detect small associations. Statistical power is also increased by analysing continuous traits rather than binary outcomes. Using this approach, more than 100 genetic loci associated with both IOP (Khawaja et al., 2018; Choquet et al., 2017), the cardinal modifiable risk factor for glaucoma, and vertical cup-disc ratio (Han et al., 2021), a marker of glaucomatous ONH damage, have been identified. Results from these analyses have shed light on the underlying pathophysiology of glaucoma (Choquet et al., 2020), while meta-analysis of this genetic data, using a multitrait approach, has further enabled POAG genetic discovery (Craig et al., 2020).

## 3. Polygenic risk scores

Although each SNP identified through GWAS explains only a small proportion of heritability and is generally insufficient to cause disease, the additive effects of multiple common variants across the genome can confer a genetic risk equivalent to that seen in monogenic disease (Khera et al., 2018). This cumulative genetic burden can be distilled into a single probabilistic value – a polygenic risk score (PRS) – that represents a quantitative summary of an individual’s genetic susceptibility to a specific trait or disease (Qassim et al., 2021). At its most basic, an unweighted PRS is a simple sum of the number of risk variants carried by an individual. More commonly, however, the variants are weighted by their magnitude of effect (based on the GWAS results), allowing for better risk prediction by accounting for both the total number of variants and the individual variant effect sizes (Chatterjee et al., 2016). In a clinical context, the utility of a PRS is not as a diagnostic tool, but rather as a means of disease risk stratification, allowing for categorisation of individuals according to their level of underlying genetic risk. Those identified to be at high risk of disease (with a PRS in the top 20% of the normal population or study cohort, for example) may then benefit from modified screening approaches or targeted interventions (Qassim et al., 2021). The clinical utility of PRS has already been reported in a host of complex non-communicable diseases, including cardiovascular disease, diabetes, and cancer (Torkamani et al., 2018).

Given the high heritability of POAG, as well as the clinical effectiveness of early interventions in preventing otherwise irreversible vision loss, the application of PRS to glaucoma risk stratification has been a research focus in recent years (Qassim et al., 2021). Early studies, generally based on a restricted set of genetic variants and applied to relatively small cohorts, were only able to demonstrate modest discriminatory powers, with limited clinical potential (Mabuchi et al., 2017; Tham et al., 2015; Zanon-Moreno et al., 2017). Backed by larger GWAS, however, recent work has been able to demonstrate risk stratification and predictive ability with clear potential for translational benefit. For example, a glaucoma PRS based on 146 IOP-associated SNPs was found to be associated with higher IOP, younger age of glaucoma diagnosis, more family members affected, and higher treatment intensity in an independent cohort (Qassim et al., 2020). More recently, a comprehensive POAG PRS, based on 2673 uncorrelated genetic variants identified using a multitrait approach, demonstrated even greater risk stratification in an independent cohort, with those in the top decile of the PRS distribution having an almost 15-fold greater risk for glaucoma relative to those in the bottom decile (Craig et al., 2020). The same PRS was also found to predict disease progression in early manifest glaucoma cases and surgical intervention in advanced disease (Craig et al., 2020; Siggs et al., 2022). Although not intended as diagnostic tools, regression-based POAG risk prediction models based on recent PRS can now achieve an area under the receiver operating characteristic curve (AUC) of 0.76,^5,19^ considered an “acceptable” level of discriminatory power for a diagnostic test (Mandrekar, 2010).

## 4. Environmental risk factors

While genetic susceptibility undoubtedly contributes a substantial proportion to individual risk, glaucoma is a complex disease and environmental determinants also play a role. Given recent advances in glaucoma PRS development, it may soon be possible to identify individuals at high risk of glaucoma before they exhibit any signs of disease, making the identification of environmental factors that could potentially modify genetic risk a particular priority.

Some factors, such as playing high-resistance wind instruments, ingesting caffeine, certain yoga positions, wearing tight neckties, and lifting weights are known to increase IOP; while others, including general physical activity and consuming alcohol, lower IOP (Pasquale and Kang, 2009). However, it is unclear whether these short-term changes are sufficient to meaningfully impact glaucoma risk, and the overall effect of habitual behaviours are less clear. Certain factors may also influence glaucoma risk through IOP-independent mechanisms by affecting the rate of RGC apoptosis (Wiggs, 2012) – various dietary factors, including antioxidants and essential fatty acids, have been implicated as potentially neuroprotective in glaucoma (Kumar and Agarwal, 2007; Al Owaifeer and Al Taisan, 2018), while others, notably alcohol, are known to be neurotoxic (Stuart et al., 2022a). A further consideration is the potential for “environmental antagonistic pleiotropism” – in which an environmental exposure may simultaneously generate biological responses that offset one another (Pasquale and Kang, 2009). However, despite extensive research and numerous reported associations, no single environmental factor has been proven as an interventional target for glaucoma in clinical trials. A brief review of the role of common environmental factors in glaucoma follows.

### Alcohol:

The short-term effects of alcohol ingestion include a transient, dose-dependent reduction in IOP (Buckingham and Young, 1986; Harris et al., 1996; Houle and Grant, 1967; Giurlani et al., 1978; Luksch et al., 2009; Peczon and Grant, 1965; Weber et al., 2013; Yamada et al., 1995) and an increase in ONH blood flow (Weber et al., 2013; Kojima et al., 2000), theoretically playing a protective role in the development of glaucoma. The effects of habitual alcohol consumption on IOP and glaucoma, however, are less clear, with several population-based studies reporting an adverse association between alcohol use and IOP (Leske et al., 1996; Lin et al., 2005; Song et al., 2020; Wu et al., 1997; Yoshida et al., 2003; Stuart et al., 2022b), although this is not always a consistent finding (Seddon et al., 1983; Weih et al., 2001). Very few studies have been designed specifically to assess the relationship between alcohol consumption and glaucoma, and while adverse associations have been reported (Stuart et al., 2022b; Wise et al., 2011), most observational studies have yielded null results (Leske et al., 1996, 2001; Bikbov et al., 2020; Bonomi et al., 2000; Charliat et al., 1994; Chiam et al., 2018; Renard et al., 2013; Jiang et al., 2012; Kang et al., 2007; Pan et al., 2017). Systematic review and meta-analysis of these studies suggests that habitual alcohol consumption is adversely related to both IOP and glaucoma, although the quality of evidence is low (Stuart et al., 2022a). Alcohol intake does appear to be consistently associated with a thinner inner retina (Stuart et al., 2022b; Khawaja et al., 2020; Lamparter et al., 2018; Paulsen et al., 2021; Han et al., 2020) – a structural characteristic of glaucoma (Kim and Park, 2018; Oddone et al., 2016) – with recent Mendelian randomisation (MR) experiments suggesting a causal relationship (Stuart et al., 2022b).

### Smoking:

Exposure to harmful compounds found in tobacco smoke has been postulated to be a risk factor for glaucoma through ischaemic or oxidative mechanisms (Jain et al., 2017b). Conversely, nicotine has been hypothesised to be a protective factor through nitric oxide-induced vasodilatory properties (Toda and Nakanishi-Toda, 2007). While acute exposure has been shown to have detrimental effects on the ocular surface and tear function (Latif and Naroo, 2022), there appears to be little short-term effect on IOP or ONH perfusion (Tamaki et al., 2000). Despite these experimental results, multiple population-based studies have reported higher IOP in smokers compared to non-smokers (Lee et al., 2003; Yoshida et al., 2014; Lee et al.), with findings from the UK Biobank suggesting that this may be related to altered corneal biomechanical properties rather than a true ocular hypertensive effect (Chan et al., 2016). The evidence for the role of smoking in glaucoma is conflicting and inconclusive. Most studies have reported null (Wise et al., 2011; Charliat et al., 1994; Juronen et al., 2000; Kang et al., 2003; Klein et al., 1993; Quigley et al., 1994; Ramdas et al., 2011; Wang et al., 2012; Wilson et al., 1987) or adverse (Renard et al., 2013; Fan et al., 2004; Kaimbo et al., 2001; Katz and Sommer, 1988; Le et al., 2003) associations, especially in current or heavy smokers (Jain et al., 2017b), but there is also evidence suggesting a potentially protective association (Buys et al., 2012; Founti et al., 2020; Doshi et al., 2008; Tran et al., 2023), despite an uncertain explanatory mechanism.

### Caffeine:

Numerous studies have examined the effect of caffeine-containing products (which include coffee, tea, carbonated drinks, and chocolate products) on ocular parameters of both healthy participants (Vera et al., 2019; Redondo et al., 2020; Terai et al., 2012; Dervişoğulları et al., 2016; Ozkan et al., 2008; Okuno et al., 2002; Ajayi and Ukwade, 2001; Lotfi and Grunwald, 1991; Okimi et al., 1991; Adams and Brubaker, 1990) and glaucoma patients (Jiwani et al., 2012; Avisar et al., 2002; Tran et al., 2014; Higginbotham et al., 1989), with most demonstrating a modest short-term elevation in IOP and reduction in ONH blood flow. Although this ocular hypertensive effect does not translate to epidemiological studies of habitual caffeine use in the general population, there does appear to be an adverse association with IOP in individuals with, or at high genetic risk for, glaucoma (Chandrasekaran et al., 2005; Kim et al., 2021). Similarly, while studies of the association between caffeine and glaucoma have reported conflicting results (Kim et al., 2021; Wu et al., 2018; Kang et al., 2008; Pasquale et al., 2012; Bae et al., 2020), an adverse relationship may only be apparent in individuals with a high genetic susceptibility to glaucoma (Kim et al., 2021; Kang et al., 2008; Pasquale et al., 2012). Recent MR experiments have provided further evidence that habitual caffeine consumption may be causally related to an increased risk for POAG (Li et al., 2022).

### Physical activity:

Bouts of physical activity are well documented to cause a transient reduction in IOP in both healthy individuals (Yan et al., 2016; Read and Collins, 2011; Avunduk et al., 1999; Ashkenazi et al., 1992; Martin et al., 1999; Natsis et al., 2009; Leighton and Phillips, 1970; Harris et al., 1994; Conte et al., 2014; Price et al., 2003; Qureshi, 1995) and glaucoma patients (Natsis et al., 2009; Qureshi, 1995), as well as an increase in ocular blood flow and perfusion of the ONH and retina (Price et al., 2003; Vo Kim et al., 2019; Alnawaiseh et al., 2017; Li et al., 2021). Fewer studies have assessed the association of habitual physical activity with IOP (Qureshi et al., 1996; Fujiwara et al., 2019) and glaucoma (Wang et al., 2019; Williams, 2009; Meier et al., 2018; Lin et al., 2017). While protective associations have been reported for both greater levels of physical activity and greater cardiovascular fitness (Qureshi et al., 1996; Fujiwara et al., 2019; Williams, 2009; Meier et al., 2018), this is not always a consistent finding in epidemiological studies (Wang et al., 2019; Lin et al., 2017; Madjedi et al., 2023).

### Diet:

There is considerable interest in the role that diet may play in modulating glaucoma risk and various individual dietary components have been studied in relation to the disease (Pasquale and Kang, 2009; Al Owaifeer and Al Taisan, 2018). Studies suggest that oxidative stress may play a role in glaucoma (Kumar and Agarwal, 2007), and many dietary factors are hypothesised to be neuroprotective through antioxidative mechanisms. These include *Ginkgo biloba* extract (which may also increase ocular blood flow and be of particular importance in NTG) (Chung et al., 1999; Hirooka et al., 2004; Eckert et al., 2005; Park et al., 2011; Shim et al., 2012; Quaranta et al., 2003; Lee et al., 2013), flavonoids (a polyphenol compound commonly found in green tea, red wine, and cocoa) (Patel et al., 2015; Kang et al., 2018), fruits and vegetables (nitrate-rich green leafy vegetables, in particular, are further hypothesised to play a role through nitric oxide signalling) (Coleman et al., 2008; Giaconi et al., 2012; Kang et al., 2016). Despite these findings, the use of antioxidant supplementation has not consistently shown a beneficial association with glaucoma (Wang et al., 2013; Garcia-Medina et al., 2015; Moreno-Montañés et al., 2022). There is also evidence that dietary niacin (vitamin B3) may be protective in glaucoma, potentially through favourable effects on neural tissue and mitochondrial function (Hui et al., 2020; Taechameekietichai et al., 2021). Dietary factors implicated as potentially harmful in glaucoma include essential fatty acids (specifically an omega-3:omega-6 imbalance) (Ren et al., 2006; Pérez de Arcelus et al., 2014; Kang et al., 2004), and excessive sodium intake (Tseng et al., 2022). Although low-carbohydrate dietary patterns, theorised to enhance mitochondrial function and have antioxidant effects (Miller et al., 2018), were not consistently associated with glaucoma in three large US prospective studies (Hanyuda et al., 2020), a combined Mediterranean and DASH (Dietary Approaches to Stop Hypertension) diet, which incorporates various individual dietary components discussed above, was recently associated with a lower risk for incident glaucoma in the Rotterdam Study (Vergroesen et al., 2023).

### Stress:

The complex systemic response associated with emotional and psychological stress has been postulated to play a role in glaucoma through various pathogenic mechanisms induced by chronic elevation and non-physiological patterns of cortisol release (Russell and Lightman, 2019). While acute stress has been shown to elevate IOP (Brody et al., 1999; Méndez-Ulrich et al., 2018; Gillmann et al., 2019; Abe et al., 2020; Kaluza et al., 1996), chronic stress and anxiety have recently been reported to be adversely associated with glaucoma (Shin et al., 2021; Berchuck et al., 2021). Stress-reduction strategies, including pharmacological and meditation-based approaches, have been shown to have beneficial effects on both IOP (Ismail and Mowafi, 2009; Dada et al., 2018, 2022) and ONH perfusion (Dada et al., 2021).

### Air pollution:

Ambient air pollution (from sources including coal combustion, automotive vehicle emissions, and biofuels) is recognised as an important contributor to the global disease burden and has been hypothesised to affect glaucoma through neurotoxic or vascular mechanisms (Cohen et al., 2017; Grant et al., 2022). Exposure to particulate matter less than 2.5 μm in diameter (PM_2.5_) has consistently been linked to a higher prevalence of glaucoma in epidemiological studies (Chua et al., 2019; Sun et al., 2021; Grant et al., 2021; Yang et al., 2021), with no evidence for an adverse association with other forms of air pollution (Grant et al., 2022).

In addition to environmental exposures, various medical conditions (Stein et al., 2021), metabolic risk factors (Roddy, 2020) and systemic medications (Wu et al., 2020) have been associated with glaucoma. A detailed overview of these relationships is beyond the scope of this review and the reader is directed to the relevant references for further information. Given the complexity of glaucoma, it is likely that these factors modulate disease risk in a similar manner to genetic factors – while the effect of individual factors may be small and generally insufficient to cause disease, the cumulative contribution of multiple factors may prove to confer appreciable risk. Although strong evidence is lacking, in general, recommendations for modifiable interventions in glaucoma can be aligned with those for overall health – appropriate management of comorbid health conditions, maintaining a normal metabolic profile, avoiding known occupational hazards, regular physical activity, a healthy diet, and effective stress management.

## 5. Gene-environment interactions

While both genetic and environmental factors can independently influence glaucoma risk, a further aetiological consideration is the interplay between the two. Studies of gene-environment interaction aim to describe how genetic and environmental factors jointly influence disease risk (Hunter, 2005). Importantly, the combined effect of gene and environment may confer a risk that reflects a departure from the simple additive effect of the two. For example, an environmental exposure may only cause an effect or be associated with a disease in the presence of a certain genetic variant (e.g., the alcohol-induced flushing response seen in individuals with low-activity polymorphisms in the *ALDH2* (aldehyde dehydrogenase 2) gene) (Edenberg, 2007). Alternatively stated, the risk of disease associated with a particular genotype may be modified by changing the level of exposure to an environmental risk factor (e.g., the risk of developing emphysema in individuals with alpha-1 antitrypsin deficiency caused by *SERPINA1* (serpin family A1) mutations can be modified by altering exposure to cigarette smoke). (Lockett et al., 2012).

Better characterisation of gene-environment interactions has several possible benefits, including offering insights into underlying biological pathways, allowing for improved public health policy through targeted population screening, and filling the missing heritability gap for complex traits (Hunter, 2005). However, despite considerable interest, studies of gene-environment interaction have historically been limited by a lack of adequately powered studies with the necessary genetic and environmental data to perform these analyses (Manolio et al., 2006). This challenge has been partially overcome by the advent of large-scale, population-based, prospective cohort studies, such as the UK Biobank (Sudlow et al., 2015; Bycroft et al., 2018), which have revolutionised epidemiological research in recent decades (Manolio et al., 2020). The increasing availability of large cohorts with detailed ophthalmic, genetic, and environmental data has allowed for greater consideration to be given to gene-environment interactions in glaucoma.

An early research focus was the role of the *NOS3* (nitric oxide synthase 3) gene in mediating glaucoma risk. The NOS3 enzyme catalyses the production of nitric oxide, which in turn influences luminal smooth muscle tone (Pollock et al., 1991). This isoform is present in the human outflow pathway and the endothelial cells of the RGC vasculature (Nathanson and McKee, 1995; Garthwaite et al., 2006), making it of interest in glaucoma. In a nested case-control study of the Nurses’ Health Study and Health Professionals Follow-up Study, while no *NOS3* polymorphism was associated with POAG overall, a significant interaction was observed between various *NOS3* SNPs and post-menopausal hormone (PMH) use in women (Kang et al., 2010). Although PMH use has previously been implicated as a protective factor in glaucoma (Madjedi et al., 2022), these findings suggest that sex-based biology and reproductive hormones may play a role in POAG pathogenesis and offer insights into potential underlying disease mechanisms. In a similar analysis, the associations of hypertension and cigarette smoking with POAG risk were also found to depend on *NOS3* genetic polymorphisms (Kang et al., 2011), again suggesting that nitric oxide signalling may play an important role in mediating the effect of environmental risk factors on glaucoma risk.

While these studies examined environmental interaction with a single genetic locus, recent advances in PRS development have now made it possible to assess the interaction between an environmental factor and the cumulative effect of multiple genetic variants. For example, it has been shown that for women in the highest decile of non-modifiable risk for breast cancer (based in part on a 92-SNP breast cancer PRS), their absolute lifetime risk could be reduced to an average level by modifying body mass index, PMH use, alcohol intake and smoking (Maas et al., 2016). Although this approach may not yield specific insights into underlying biological pathways, it may have important implications for targeted population screening and personalised recommendations for primary preventative measures.

Recently, the same approach has been applied to the study of glaucoma-related gene-environment interactions. In a study of more than 100,000 UK Biobank participants, caffeine consumption was found to be associated with both higher IOP and glaucoma prevalence, but only in those at the highest genetic susceptibility to higher IOP (based on a 111-SNP IOP PRS) (Khawaja et al., 2018; Kim et al., 2021). Specifically, among those with a PRS in the top 25% of the study population, consuming >480 mg/day versus <80 mg/day of caffeine was associated with a 0.35-mmHg higher IOP (Kim et al., 2021). Although this population-level difference may appear small, it is equivalent in magnitude to the effect of *TMCO1* rs10918274, the gene variant with the strongest effect on both higher IOP and POAG risk (Khawaja et al., 2018).

In a similar UK Biobank analysis, alcohol consumption was demonstrated to be adversely associated with IOP, but again only in those with the greatest genetic risk for glaucoma (based on the previously described 2673-SNP multitrait glaucoma PRS) (Craig et al., 2020; Stuart et al., 2022b). While no association between alcohol intake and IOP was observed in those with a PRS in the bottom 20% of the distribution, progressively stronger associations were noted in those at higher genetic risk, with those in the top 20% having 0.15-mmHg higher IOP per standard deviation greater weekly alcohol intake (Stuart et al., 2022b). Together, these results provide preliminary evidence that individuals with a high genetic susceptibility to glaucoma may be able to meaningfully reduce their absolute risk of disease by modifying their exposure to two common dietary factors.

## 6. Discussion

Until recently, technical and financial considerations have largely restricted complex disease genetics and PRS application to the realm of scientific research; however, rapid advances in the field have now placed these firmly in the public domain. Many commercial enterprises (e.g., 23andMe, www.23andme.com) now offer publicly-accessible genotyping services and provide a range of personalised health insights – including PRS calculation – based on an individual’s genetic data. Ambitious projects, such as Our Future Health (www.ourfuturehealth.org.uk) which aims to genotype 5 million UK adults by 2025, provide further indication that population-scale genotyping is fast becoming a distinct reality. As we approach a future where individual genetic data and polygenic risk are readily available, knowledge of gene-environment interactions will become increasingly relevant to the management of glaucoma and has two important fundamental implications:

### Primary prevention:

While the onset of POAG typically occurs from middle-age onwards, any underlying genetic predisposition to the disease is fixed from birth. Applied to a sufficiently young population, a PRS could identify those individuals at high risk for developing glaucoma, but before the overt onset of disease. While these individuals may benefit from targeted screening and regular ophthalmic examinations (population-level screening for glaucoma is currently not recommended (Stein et al., 2021)), it also raises the possibility of primary disease prevention – high-risk individuals could delay, and potentially prevent, the onset of disease by modifying environmental exposures. Genetic risk stratification with subsequent environmental modification, including physical activity and statin therapy, in disease-free individuals has already been suggested as a primary preventative measure for coronary artery disease (Roberts et al., 2021). This paradigm shift in complex disease management has the potential to revolutionise preventative medicine in the future.

### Personalised medicine:

As knowledge of gene-environment interactions in glaucoma improves, so will the ability to provide individualised glaucoma management. In future, genetic profiles could guide targeted environmental recommendations, allowing high-risk individuals to modify their absolute disease risk and providing them greater autonomy in the management of their health. This personalised approach may also aid a physician’s glaucoma management plan. Genetic data may guide optimal therapeutic choices (e.g., by predicting which individuals will respond to particular ocular hypotensive medication classes or laser therapy) and could lead to improved clinical outcomes and more efficient use of limited healthcare resources. The use of pharmacogenetics to guide treatment options has recently been advised for several common medications, including opioids and antidepressants, in the UK (Mahase, 2022), and it is not implausible that this approach may eventually prove to become common practice when prescribing glaucoma therapeutics in future.

Further discovery of glaucoma-associated genetic variants and environmental risk factors, coupled with ongoing advances in analytical tools to explore gene-environment interactions (Westerman et al., 2021), promise to greatly improve our understanding of this complex disease and enable novel disease management strategies in years to come.
